# Tet-Inducible Production of Infectious Zika Virus from the Full-Length cDNA Clones of African- and Asian-Lineage Strains

**DOI:** 10.3390/v10120700

**Published:** 2018-12-09

**Authors:** Lizhou Zhang, Wei Ji, Shuang Lyu, Luhua Qiao, Guangxiang Luo

**Affiliations:** 1 Department of Microbiology, University of Alabama at Birmingham School of Medicine, Birmingham, AL 35294, USA; lizhou8728@gmail.com (L.Z.); shuanglv@uab.edu (S.L.); luhuaq@gmail.com (L.Q.); 2 Department of Microbiology, Peking University Health Science Center School of Basic Medical Sciences, Beijing 100191, China; jiwei_yunlong@126.com

**Keywords:** Zika virus, reverse genetics, infectious cDNA, Tet-inducible, MR766, FSS13025

## Abstract

Zika virus (ZIKV) is a mosquito-borne flavivirus that has emerged as an important human viral pathogen, causing congenital malformation including microcephaly among infants born to mothers infected with the virus during pregnancy. Phylogenetic analysis suggested that ZIKV can be classified into African and Asian lineages. In this study, we have developed a stable plasmid-based reverse genetic system for robust production of both ZIKV prototype African-lineage MR766 and clinical Asian-lineage FSS13025 strains using a tetracycline (Tet)-controlled gene expression vector. Transcription of the full-length ZIKV RNA is under the control of the Tet-responsive *P*_tight_ promoter at the 5′ end and an antigenomic ribozyme of hepatitis delta virus at the 3′ end. The transcription of infectious ZIKV RNA genome was efficiently induced by doxycycline. This novel ZIKV reverse genetics system will be valuable for the study of molecular viral pathogenesis of ZIKV and the development of new vaccines against ZIKV infection.

## 1. Introduction

Zika virus (ZIKV), an arthropod-borne virus, belongs to the *Flavivirus* genus of the Flaviviridae family, which includes several other important human pathogens such as yellow fever virus (YFV), dengue virus (DENV), and West Nile virus (WNV). ZIKV was first discovered in the Zika forest area of Uganda in 1947 [1]. In recent years, it has emerged as an important human viral pathogen, causing several major epidemics in Yap Island and Micronesia, French Polynesia, and South America [2]. Although ZIKV infection is often asymptomatic or mild in clinical manifestation, it can cause congenital malformations, including microcephaly, intrauterine growth retardation, and neurodevelopmental delays, in infants born to mothers infected with the virus during pregnancy [3]. Its infection is also associated with the neurologic disorder Guillain–Barre syndrome in adults [4]. In contrast to other mosquito-borne flaviviruses, ZIKV can cause persistent infection in humans and primates [5,6] and can be transmitted sexually [7,8,9]. Currently, there are no antiviral drugs or vaccines for the control of ZIKV infection.

ZIKV is an enveloped RNA virus with a positive-sense and single-stranded RNA genome of about 10,800 nucleotides. The viral genomic RNA contains a single open reading frame encoding a large viral polyprotein of 3423 amino acids. Upon translation, the viral polyprotein is cleaved by host and viral proteases into structural proteins (capsid [C], premembrane [prM], and envelope [E]) and nonstructural (NS) proteins (NS1, NS2A, NS2B, NS3, NS4A, NS4B, and NS5) [10,11]. Phylogenetic analysis classified ZIKV into two major lineages: African lineage and Asian lineage, which exhibit different growth capacity and virulence in vitro and in vivo [12,13,14,15]. However, the underlying molecular aspects for distinct viral growth and pathogenesis between African and Asian lineages remain unknown.

The recent development of ZIKV reverse genetics systems has made it possible to determine the role and underlying molecular mechanism of viral proteins in viral replication and pathogenesis as well as for attenuated virus vaccine development [16,17,18,19,20,21]. Over the course of ZIKV cDNA construction, its genetic instability and toxicity in bacteria were found to be major obstacles to obtaining the full-length infectious cDNA, particularly for the prototype MR766 virus. It was speculated that the presence of cryptic bacterial promoters permits expression of viral peptides/proteins that are toxic to bacteria [22,23,24]. Several strategies have been explored to overcome the problems associated with viral toxicity and genetic instability in bacteria. The first full-length cDNA of ZIKV (Asian-lineage FSS13025 strain) was successfully constructed by using a very-low-copy plasmid as the vector in conjunction with a T7 promoter for in vitro transcription of ZIKV RNA by a T7 RNA polymerase [18]. Similarly, another group used an SP6 promoter to drive in vitro transcription of the full-length ZIKV RNA. In the latter case, a self-splicing intron was inserted into the C-terminal coding region of the E protein. The SP6 RNA transcripts were subjected to in vitro splicing in order to produce the infectious ZIKV RNA [25]. Alternatively, ZIKV cDNA fragments were amplified separately and cloned into different plasmids, followed by Gibson assembly or DNA ligation in vitro to produce the full-length ZIKV cDNA [20,21,26]. The resulting full-length cDNA was subsequently used as the template for in vitro T7 transcription to produce infectious ZIKV RNAs. Apart from in vitro transcription of infectious ZIKV RNA, infectious ZIKV RNA could be directly produced in the cell by cloning its cDNA into a mammalian expression vector. Transcription of infectious ZIKV RNA is driven by a polymerase II promoter at the 5′ end of ZIKV cDNA. In the case of infectious ZIKV cDNA, a synthetic intron sequence had to be inserted into the *NS1* or *NS5* gene in order to avoid viral toxicity to bacteria [16,19]. The silence of the cryptic bacterial promoters was also reported to get around toxicity [23]. In present study, we have constructed infectious ZIKV cDNA independent of in vitro RNA transcription or intron insertion using a tetracycline (Tet)-controlled transcription vector. The full-length ZIKV cDNAs were cloned into the pTRE-Tight vector under the control of the Tet-responsive *P*_tight_ promoter and the antigenomic ribozyme of hepatitis delta virus (HDV). Both African- and Asian-lineage ZIKV were robustly produced upon DNA transfection and treatment with doxycycline. This novel ZIKV reverse genetics system will facilitate genetic determination of the underlying molecular mechanism of ZIKV replication and pathogenesis as well as genetic manipulation of infectious ZIKV for vaccine development.

## 2. Materials and Methods 

### 2.1. Cell Culture and Virus

Vero and C6/36 (CRL-1660) cell lines were obtained from America type culture collection (ATCC, Manassas, VA, USA) and cultured in Dulbecco’s modified Eagle’s medium (DMEM) supplemented with 10% fetal bovine serum (FBS) (Atlanta Biologicals, Atlanta, GA, USA), 0.1 mM nonessential amino acids, penicillin-streptomycin (Sigma-Aldrich, St. Louis, MO, USA and HyClone, Logan, UT, USA) at 37 °C in a 5% CO_2_ incubator. ZIKV African-lineage MR766 strain was obtained from ATCC and propagated in C6/36 and Vero cells.

### 2.2. DNA Construction 

pTRE-Tight vector containing the Tet-responsive element and a minimal cytomegalovirus (CMV) promoter (*P*_tight_) was described previously [27]. pTet-On vector expressing a reverse Tet-responsive transcriptional activator (rtTA) was from Takara Bio. The full-length infectious cDNA of ZIKV/MR766 was amplified by reverse transcription polymerase chain reaction (RT-PCR) similar to the methods used previously by others [16,18]. The virion RNA (vRNA) was isolated from the supernatant of the ZIKV/MR766-infected Vero cells using QIAamp Viral RNA Kits (Qiagen, Inc., Valencia, CA, USA). The full-length viral cDNA was initially amplified as five subgenomic cDNA fragments by RT-PCR using specific primers containing unique restriction enzyme sites and cloned into the p*EASY*-Blunt vector (Transgen Biotech, Beijing, China), resulting in five subgenomic cDNA clones, which were confirmed by DNA sequence analysis. These five cDNA fragments were sequentially inserted into a modified pTRE-Tight vector that allows inducible transcription of infectious ZIKV RNA in the cell. Initially, the pTRE-Tight vector was modified by replacing the high-copy-number ColE1 origin of replication with the low-copy-number p15A origin of replication from the vector pACYC177 [18]. For construction of infectious ZIKV/MR766 cDNA, a DNA fragment containing a unique *Sbf* I restriction enzyme site, partial 3′ end sequence (nucleotides 10,779–10,807 with a unique *Eag* I site) of the MR766 genome, and the antigenomic ribozyme of HDV were amplified by PCR and introduced into the low-copy pTRE-Tight vector between the restriction enzyme sites *Sac* I and *Xba* I. Lastly, the nucleotides 1 to 1711 of the MR766 cDNA were fused with the minimal CMV promoter sequence by PCR using synthetic primers containing unique *Sac* I and *Sbf* I sites and inserted into the above modified pTRE-Tight vector, resulting in the pTight-ZIKV/MR766 entry vector. For subsequent cloning of other cDNA fragments excised from subgenomic cDNA clones, partial Fragment 1 between *EcoR* I and *Age* I sites and Fragment 2 between *Age* I and *Sbf* I sites were simultaneously inserted into the pTight-ZIKV/MR766 Entry Vector digested with restriction enzymes *EcoR* I and *Sbf* I. The rest of the three cDNA fragments (3, 4, and 5) were then cloned into the *Sbf* I/*Eag* I-digested pTight-ZIKV/MR766 Entry vector containing Fragments 1 and 2, resulting in a full-length MR766 cDNA clone. Similarly, the Asian-lineage ZIKV strain FSS13025 cDNA was cloned into the pTRE-Tight vector, resulting in pTight-ZIKV/FSS13025. The full-length FSS13025 cDNA (GenBank number KU955593.1) was synthesized and cloned into the vector pCCI-Brick by GenScript, resulting in a plasmid designated pCCI-Brick-ZIKV_FSS13025. The FSS13025 cDNA between restriction enzyme sites *Nhe* I and *Eag* I was excised from the synthetic pCCI-Brick-ZIKV_FSS13025 vector and inserted into the pTight-ZIKV/FSS13025 entry vector cut by the same *Nhe* I and *Eag* I enzymes. The pTight-ZIKV/FSS13025 entry vector was modified from the pTight-ZIKV/MR766 Entry vector by replacing the DNA fragment between restriction enzyme sites *Sac* I and *Eag* I with a synthetic DNA fragment containing part of the minimal CMV promoter and 5′ end nucleotides 1–57 of the FSS13025 genome. DNA ligation was carried out by incubation of the above-described DNA vectors and inserts with T4 DNA ligase (NEB, M0202) at 16 °C overnight. The ligated DNA was concentrated by ethanol precipitation and resuspended in distilled water prior to DNA transformation. One Shot TOP10 Electrocomp *Escherichia coli* (*E. coli)* was from Invitrogen (cat. no. C404052, Carlsbad, CA, USA) and used for DNA transformation by electroporation, which was carried out in a 2 mm cuvette in the following conditions: 2500 V, 25 μF, 200 Ω. The DNA-transformed *E. coli* was spread onto lysogeny broth (LB) plate containing 100 μg/mL of Ampicillin with about 20-h incubation at 30 °C. The clones were cultured in LB medium by shaking (170 rpm) at 30 °C overnight. Plasmid DNA was extracted using QIAGEN kits and confirmed by DNA sequence analysis. 

### 2.3. DNA Mutagenesis

To engineer a genetic marker for rescued MR766 virus, the restriction enzyme *Nhe* I site at the nucleotide 3862 of the MR766 cDNA was mutated by an overlapping PCR method using synthetic oligonucleotide primers containing nucleotide mutations that did not change amino acid sequence (silent mutation). The PCR products were digested by *Sal* I and *Sbf* I and inserted into the infectious cDNA clone pTight-ZIKV/MR766 which was similarly cut by *Sal* I and *Sbf* I. Likewise, the *Nsi* I site at the nucleotide 7178 of the FSS13025 cDNA was destroyed by introducing silent mutations into the DNA fragment between *Kas* I and *Afl* II sites of the infectious FSS13025 cDNA vector pTight-ZIKV/FSS13025.

### 2.4. DNA Transfection and Virus Production

Vero cells were seeded in a 12-well plate at a density of 2.5 × 10^5^ per well and cultured in DMEM containing 10% FBS overnight. After washing with 1× Phosphate-buffered saline (PBS) twice, Vero cells were transfected with 1 μg of pTight-ZIKV/MR766 or pTight-ZIKV/FSS13025 DNA with 1 μg of either pTRE-Tight or pTet-On vector DNA in Opti-MEM containing 4 μL lipofectamine 2000 (Invitrogen, cat. no. 11668019, Carlsbad, CA, USA). At 4 h post-transfection (p.t.), Opti-MEM was replaced with 1 mL of DMEM containing 3% FBS and 1 μg/mL of doxycycline (Sigma, D9891). The supernatants from DNA-transfected Vero cells were collected at 3 (MR766) or 6 (FSS13025) days after DNA transfection for determining the titers of infectious ZIKV.

### 2.5. Immunofluorescence Assay (IFA)

The expression of ZIKV proteins in the DNA-transfected or virus-infected Vero cells was determined by immunofluorescence assay (IFA) using a monoclonal antibody specific to the envelope protein (EMD Millipore, clone D1-4G2-4-15, Billerica, MA, USA). The DNA-transfected or virus-infected Vero cells were fixed with methanol at −20 °C for 15 min and then blocked with a PBS buffer containing 1% FBS, 1% BSA, and 0.05% Tween-20 at room temperature (RT) for 1 h. Cells were then incubated with the E monoclonal antibody at RT for 2 h, followed by incubation with a goat anti-mouse secondary antibody conjugated with Alexa Fluor 488 (Life Technologies, cat. no. A21202, Carlsbad, CA, USA) at RT for 1 h. The cell nucleus was stained with Hoechst 33342 at RT for 10 min. Fluorescent images were captured using Nikon Eclipse Ti microscope.

### 2.6. Virus Titration and Plaque Assay

A plaque assay was used to determine the titer of infectious ZIKV recovered from Vero cells transfected with infectious ZIKV cDNA clones as described above. Infectious ZIKV in the cell culture supernatant was titrated by a 10-fold serial dilution. Vero cells in 12-well cell culture plates were infected with 10-fold serially diluted ZIKV in triplicate. The virus-infected cells were incubated at 37 °C for two hours with gentle swirling several times. The virus-infected cells were then grown in DMEM containing 5% of FBS and 1% of methyl cellulose at 37 °C. At 4 to 6 days post-infection, the virus-infected cells were fixed with 4% formaldehyde solution at RT for 1 h. Viable cells were stained with 1% crystal violet solution at RT for 15 min. The numbers of plaques were manually counted and converted to infectious virus titers per milliliter.

### 2.7. Determination of the Stability of Infectious Zika Virus (ZIKV) cDNA Clone 

To determine the stability of infectious ZIKV cDNA clones pTight-ZIKV/MR766 and pTight-ZIKV/FSS13025 in *E. coli*, the plasmid DNA was amplified in TOP10 Electrocomp *E. coli* by several rounds of DNA transformation and amplification. Purified plasmid DNA after each round of amplification was initially validated by restriction enzyme digestion to determine the sizes of plasmid DNA. Purified plasmid DNA after each round of transformation and amplification was also used for DNA transfection into Vero cells to determine its ability to produce infectious ZIKV.

### 2.8. Statistical Analysis

Graphical representation and statistical analyses were performed by Prism6 software (GraphPad Software, La Jolla, CA, USA). Mean values and standard deviation (SD) were calculated from at least three independent experiments. Comparisons between samples were done using the Students *t*-test. *p* < 0.05 was considered statistically significant.

## 3. Results

### 3.1. Construction of the Full-Length Infectious ZIKV cDNA

**Figure 1:**
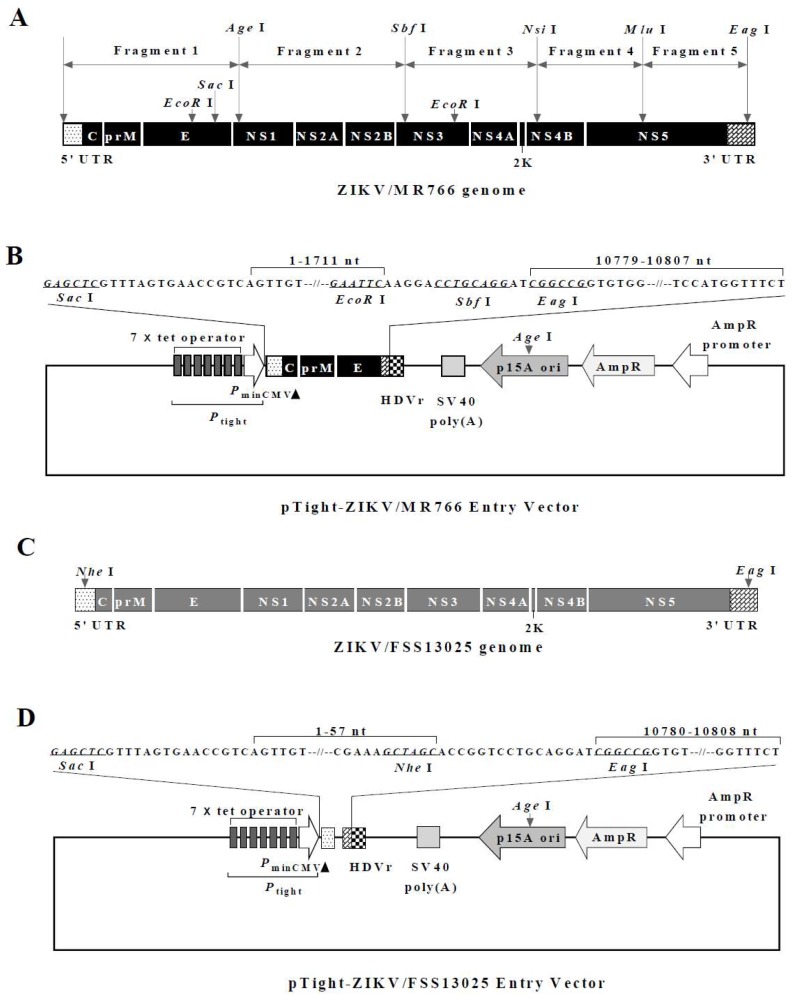
Schematic diagrams of the infectious Zika virus (ZIKV) cDNA amplification and cloning. (**A**) Illustration of subgenomic cDNA amplification and cloning of the African-lineage ZIKV/MR766 strain. Five subgenomic cDNA fragments (1 to 5) of the full-length MR766 genome were initially amplified from its vRNA by reverse transcription polymerase chain reaction (RT-PCR) using specific primers containing unique restriction enzyme sites highlighted in bold on the top. (**B**) Diagram of the pTight-ZIKV/MR766 entry vector. The entry vector was modified from pTRE-Tight by replacing the high-copy-number ColE1 origin of replication with the low-copy-number p15A origin and then inserting the partial MR766 genomic sequence with *EcoR* I, *Sbf* I and *Eag* I sites and hepatitis delta virus (HDV) antigenomic ribozyme sequence at the multiple clone sites region. The rest of the subgenomic cDNA fragments were sequentially inserted into the pTight-ZIKV/MR766 entry vector through the unique enzyme sites, resulting in an infectious MR766 cDNA clone designated pTight-ZIKV/MR766. (**C**) Diagram of the FSS13025 ZIKV genome organization. Two unique restriction enzyme sites *Nhe* I and *Eag* I at the 5′ and 3′ ends are highlighted in bold on the top. (**D**) Schematic map of the pTight-ZIKV/FSS13025 entry vector. A DNA fragment containing the 3′ end 21 nucleotides of the minimal CMV promoter, the 5′ end 57 nucleotides and the 3′ end 29 nucleotides of the FSS13025 cDNA with a 16-nucleotides spacer, and the HDV antigenomic ribozyme were inserted into the low-copy-number pTRE-Tight vector, resulting in the pTight-ZIKV/FSS13025 entry vector. The FSS13025 cDNA between *Nhe* I and *Eag* I sites was released from the full-length FSS13025 cDNA vector pCCI-Brick-ZIKV_FSS13025 (synthesized by GenScript) and cloned into the pTight-ZIKV/FSS13025 entry vector, resulting in an infectious FSS13025 cDNA clone designated pTight-ZIKV/FSS13025.

Genetic instability of Flavivirus cDNA in bacteria is a common problem encountered during the course of developing reverse genetics for certain members of the Flaviviridae family. The full-length cDNA clones of different ZIKV strains have been constructed recently by others using a T7/SP6 transcription vector or a mammalian expression vector that requires the insertion of intron sequence into the NS1 or NS5 gene in order to obtain the infectious cDNA clones [16,18,19,20,21]. In the present study, we sought to develop a more convenient and robust reverse genetics system for inducible production and genetic manipulation of infectious ZIKV of various lineages independent of in vitro T7/SP6 polymerase transcription or insertion of intron sequence using a strategy similar to our previous work on the hepatitis C virus (HCV) [28]. The construction of infectious cDNAs of MR766 and FSS13025 strains is described in materials and methods. The full-length cDNA of MR766 was sequentially spliced from 5 subgenomic cDNAs (Figure 1A) and cloned into pTight-ZIKV/MR766 entry vector (Figure 1B), whereas the full-length cDNA of FSS13025 strain was chemically synthesized by GenScript (Figure 1C) and cloned into pTight-ZIKV/FSS13025 entry vector (Figure 1D). The transcription of the full-length ZIKV RNA genome is under the control of the Tet-responsive *P*_tight_ promoter, and the HDV antigenomic ribozyme is placed immediately downstream of the 3′ end of ZIKV RNA genome (Figure 1B). Since *P*_tight_ promoter contains 7× repeat tetracycline responsive operator, the ZIKV RNA transcription would be driven by the binding of reverse Tet-responsive transcriptional activator (rtTA) to the operator in the presence of tetracycline or its derivatives doxycycline. Upon co-transfection of pTight-ZIKV with pTet-On which express rtTA and the addition of doxycycline, the full-length ZIKV RNA genome is transcribed and further processed by the HDV ribozyme-mediated cleavage at the 3′ end, resulting in an infectious RNA with precise 5′ and 3′ ends in the cell. This vector design was successfully used for the construction of the full-length cDNAs of both African-lineage MR766 and Asian-lineage FSS13025 strains of ZIKV, which exhibited genetic instability in bacteria as reported by others [16,18]. 

### 3.2. Tet-Inducible Production of Infectious ZIKV from Its cDNA

**Figure 2:**
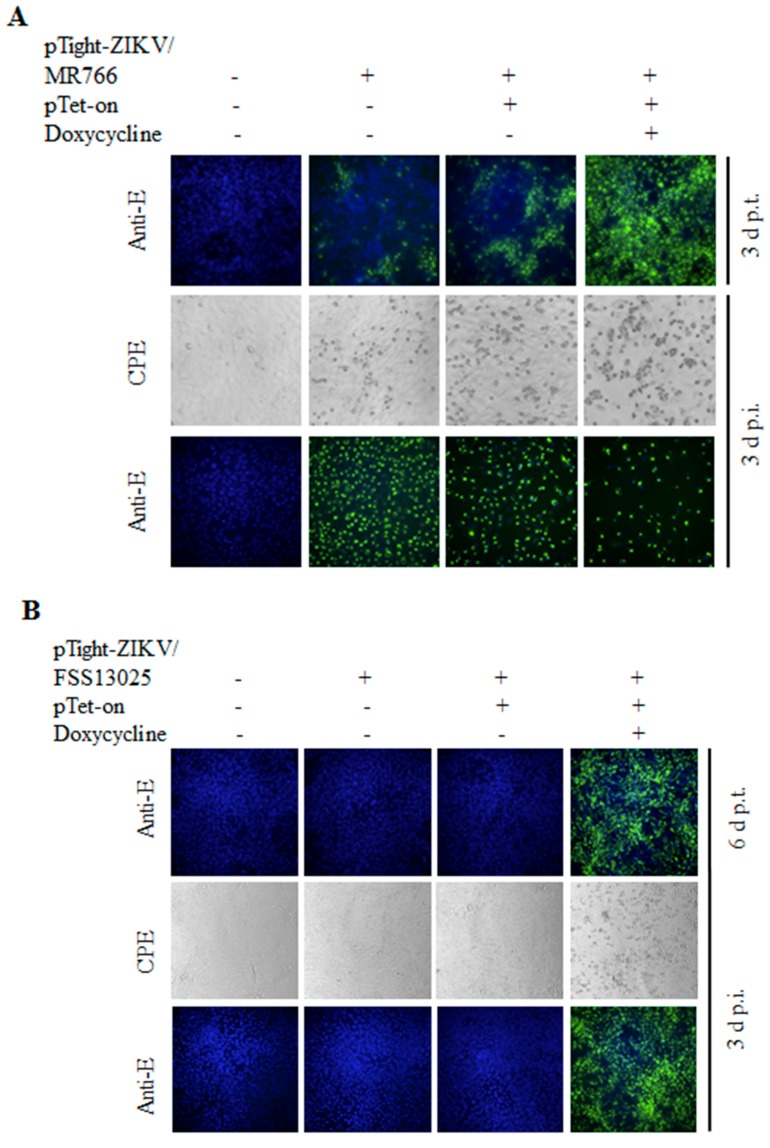
Production of cDNA-derived infectious ZIKV. (**A**) Production of infectious ZIKV/MR766 virus upon DNA transfection with or without Tet-induction. Vero cells (2.5 × 10^5^/well) in 12-well cell culture plates were transfected with 1 μg of pTight-ZIKV/MR766 DNA and 1 μg of empty vector or pTet-On vector using lipofectamine 2000. At 3 days p.t., the viral E protein in the DNA-transfected cells was detected by immunofluorescence assay (IFA) using an E-specific monoclonal antibody (D1-4G2-4-15). At the same time, the supernatants were used to infect fresh Vero cells. At 3 days p.i., the cytopathic effect (CPE) was recorded and the E protein in the ZIKV/MR766-infected cells was determined by IFA. (**B**) Determination of cDNA-derived ZIKV/FSS13025 replication and production by CPE and IFA. Experiments were carried out in the same way as in (**A**) except that the pTight-ZIKV/FSS13025 DNA was used. At 6 days p.t., the E protein was detected by IFA in the pTight-ZIKV/FSS13025 DNA-transfected Vero cells. The ZIKV/FSS13025 in the supernatant was used to infect Vero cells. At 3 days p.i., CPE was photographed and the E protein was measured by IFA as described in (**A**). Images in (**A**,**B**) were 200× magnification.

To demonstrate the functionality of the full-length ZIKV cDNA constructs described above, we have carried out DNA transfection experiments and determined the production of infectious ZIKV. We also sought to determine the Tet-inducible production of infectious ZIKV by co-transfection of Vero cells with both pTight-ZIKV/MR766 and pTet-On with and without the addition of doxycycline, a tetracycline analogue. Initially, the pTight-ZIKV/MR766 DNA was transfected into Vero cells with or without co-transfection with the pTet-On vector. The DNA-transfected Vero cells were then cultured in DMEM medium with or without doxycycline (1 μg/mL). At 3 days post-transfection (p.t.), the cytopathic effect (CPE) could be observed in the DNA-transfected Vero cells when doxycycline was added. The cell culture supernatants were harvested for the determination of infectious ZIKV, whereas the DNA-transfected cells were fixed and stained with a monoclonal antibody specific to the ZIKV E protein (Figure 2A). The expression of viral E protein was detected in cells transfected with the pTight-ZIKV/MR766 DNA regardless of co-transfection with the pTet-On vector or presence of doxycycline, suggesting leaky transcription from the *P*_Tight_ promoter (Figure 2A). However, the number of E-positive cells and the level of E protein expression were the highest in the cells co-transfected with the pTet-On vector and cultured in the presence of doxycycline (top-right corner, Figure 2A), demonstrating Tet-inducible expression of ZIKV RNA from its cDNA vector. Likewise, infectious ZIKV in the cell culture supernatant was detected by IFA (bottom images of Figure 2A). At 3 days post-infection (p.i.), CPE was observed in Vero cells infected with the cell culture supernatants from the pTight-ZIKV/MR766 DNA-transfected cells (middle panels of Figure 2A). Again, infectious ZIKV in the supernatant of the cells co-transfected with pTight-ZIKV/MR766 and pTet-On in the presence of doxycycline resulted in the highest level of CPE. Similarly, the E protein was detected by IFA staining in the ZIKV-infected cells (bottom panel of Figure 2A). It should be noted that fewer E-positive cells observed in the cells infected with the supernatants from Vero cells co-transfected with both pTight-ZIKV/MR766 and pTet-On were due to cell death caused by more infectious virus (detached from the plate). Taken together, these findings demonstrate that the full-length cDNA of the African-lineage MR766 strain is able to produce infectious virus in a Tet-inducible manner. 

Similar to MR766 cDNA clone, the Asian-lineage FSS13025 cDNA also resulted in the expression of E protein and production of infectious virus upon DNA transfection into Vero cells although at a much later time (6 days post-transfection), as determined by IFA (Figure 2B). In contrast to MR766 cDNA, transfection with FSS13025 cDNA per se failed to express the E protein or produce infectious virus. The expression of E protein and the production of infectious virus were only detected in Vero cells co-transfected with pTight-ZIKV/FSS13025 and pTet-On DNAs in the presence of doxycycline (Figure 2B). CPE and E protein could only be seen in Vero cells infected with the cell culture supernatant derived from cells co-transfected with pTight-ZIKV/FSS13025 and pTet-On in the presence of doxycycline (Figure 2B). These results suggest that the transcription and expression of infectious FSS13025 RNA from its cDNA clone in the cell are highly dependent on the expression of rtTA and the presence of doxycycline.

**Figure 3:**
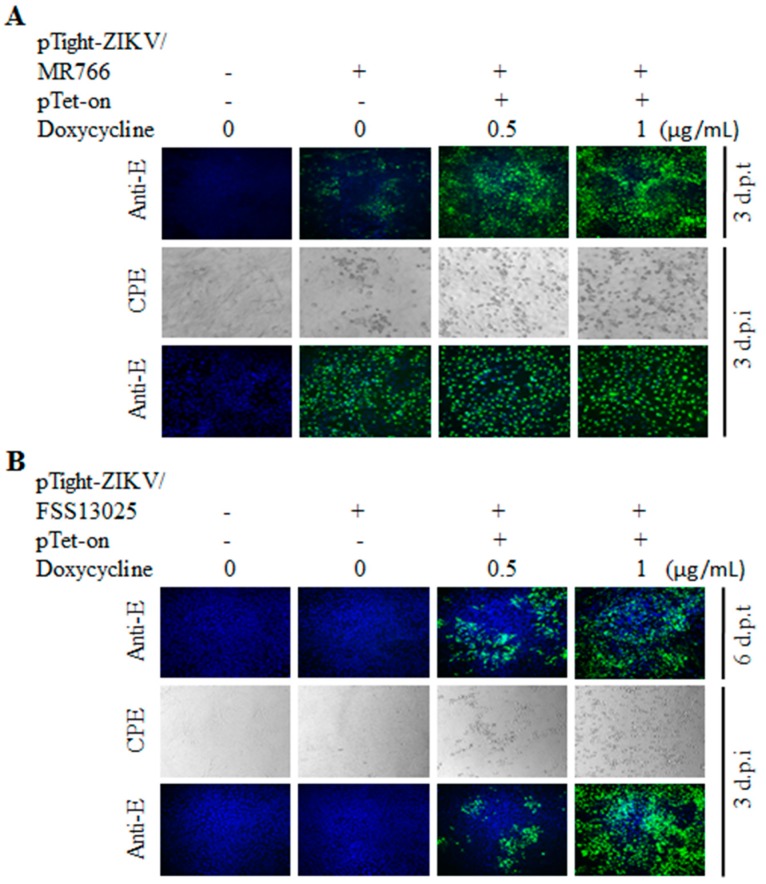

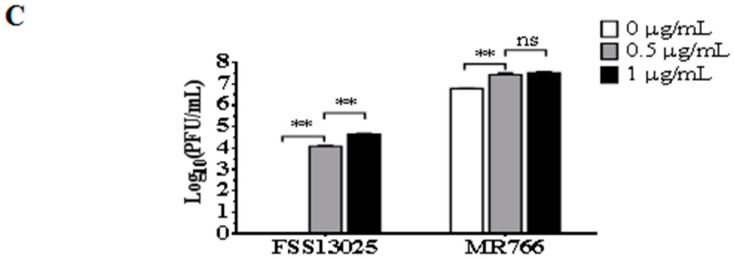
Doxycycline dose-dependent production of cDNA-derived ZIKV. Vero cells in 12-well plates were co-transfected with 1 µg of pTight-ZIKV/MR766 or pTight-ZIKV/FSS13025 DNA and 1 µg of an empty vector or pTet-On. The DNA-transfected Vero cells were cultured with different concentrations (0, 0.5, and 1 µg/mL) of doxycycline. The viral E protein in the DNA-transfected cells was determined by IFA at 3 d p.t. (for MR766) or 6 d p.t. (for FSS13025). The supernatants harvested at 3 d p.t. (MR766) or 6 d p.t. (FSS13025) were used to infect naïve Vero cells. CPE formation was recorded and the E protein was detected by IFA in the virus-infected cells at 3 d p.i. Infectious virus titers were quantified by a limiting dilution and plaque assay in the same way as Figure 3. (**A**) Determination of the E protein in Vero cells co-transfected with pTight-ZIKV/MR766 DNA and vector or pTet-On or infected with cDNA-derived ZIKV/MR766 virus by IFA. CPE formation was also documented in the cDNA-derived ZIKV/MR766 virus. (**B**) IFA detection of the E protein in Vero cells co-transfected with pTight-ZIKV/FSS13025 and pTet-On DNAs or infected with cDNA-derived ZIKV/FSS13025 virus. CPE formed by the cDNA-derived ZIKV/FSS13025 virus is shown in the middle. The images of (**A**,**B**) were taken under 200× magnification. (**C**) Doxycycline dose-dependent production of cDNA-derived ZIKV between the pTight-ZIKV/MR766 and pTight-ZIKV/FSS13025 DNAs. The supernatants from the DNA-transfected Vero cells as described in (**A**,**B**) were 10-fold serially diluted and were used for plaque assay. Infectious ZIKV titers were converted from plaque numbers and calculated as plaque-forming units per milliliter (PFU/mL). Values represent the means ± standard deviations (SD) from three independent experiments. Statistical significance was analyzed by Student’s *t*-test: ** *p* < 0.01. ns indicates no significant difference.

To further determine the Tet-inducible expression and production of infectious ZIKV from its cDNA, Vero cells were co-transfected with the pTight-ZIKV/MR766 or pTight-ZIKV/FSS13025 and pTet-On with addition of varying concentrations (0, 0.5, and 1 µg/mL) of doxycycline. The expression of the E protein in the DNA-transfected or virus-infected cells was determined by IFA using an E-specific monoclonal antibody. CPE formation in the virus-infected cells was documented by photography under an optical microscope. Indeed, the E protein expression in the pTight-ZIKV/MR766 DNA-transfected cells was significantly enhanced by increasing concentrations of doxycycline (top images, Figure 3A). CPE in the supernatant-infected cells was also increased in proportion to doxycycline concentrations. More importantly, the titers of infectious ZIKV/MR766 virus were significantly higher when doxycycline was added to the cell culture medium (Figure 3C). More significantly, the E protein expression and infectious virus production from the ZIKV/FSS13025 cDNA were strictly dependent on the presence of doxycycline in a dose-dependent manner (Figure 3B,C). Collectively, these findings demonstrate the Tet-inducible production of infectious ZIKV from its cDNA under the control of a Tet-responsive promoter, especially for ZIKV with less replication efficiency like the Asian-lineage FSS13025 strain.

### 3.3. Comparison of Virus Growth between the cDNA-Derived MR766 and FSS13025 Viruses 

**Figure 4:**
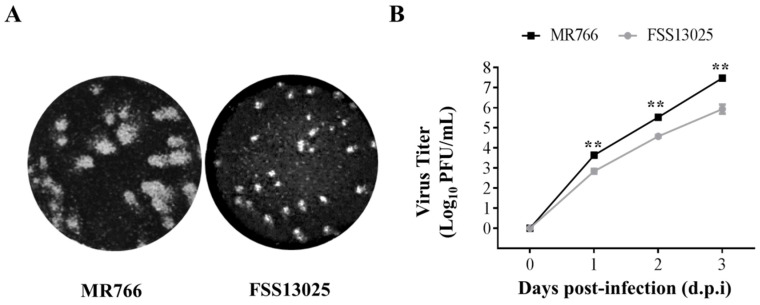
Comparison of cDNA-derived MR766 and FSS13025 virus growth. Vero cells (2.5 × 10^5^/well) in 12-well plates were co-transfected with 1 µg pTight-ZIKV/MR766 or pTight-ZIKV/FSS13025 DNA and 1 µg pTet-On vector, followed by the addition of 1 µg/mL Doxycycline to cell culture media. Infectious virus titers in the supernatants collected at day 3 (MR766) or day 6 (FSS13025) were quantified by a plaque assay. (**A**) Plaque formation by cDNA-derived ZIKV/MR766 and ZIKV/FSS13025 viruses. Vero cells in 12-well cell culture plate were infected with 30 PFU of either MR766 or FSS13025. Plaques were stained and visualized after 4 days (for MR766) or 6 days (for FSS13025) p.i. (**B**) Growth curves of cDNA-derived MR766 and FSS13025 viruses. To compare the growth ability between MR766 and FSS13025, 4 × 10^5^ Vero cells seeded in 6-well plates were infected with MR766 or FSS13025 virus at 0.01 MOI at 37 °C for 1 h. Upon washing with phosphate-buffered saline (PBS) three times, infected Vero cells were incubated with 2 mL DMEM containing 10% FBS. At day 1, 2, and 3 post-infection, virus in the supernatant was collected and stored at −80 °C. Virus yields at different time points were determined by a plaque assay. Data points represent the mean titer of triplicates. Statistical significance was analyzed by Student’s *t*-test: ** *p* < 0.01.

Transfection of pTight-ZIKV/MR766 cDNA into Vero cells resulted in higher titers of infectious virus than that of pTight-ZIKV/FSS13025 cDNA (Figure 3C), suggesting that MR766 grows more efficiently than the FSS133025 virus. To compare their growth capacity in Vero cells, we have determined the plaque-forming ability and growth curves of the cDNA-derived MR766 and FSS13025 ZIKVs. As shown in Figure 4A, MR766 virus formed bigger plaques than FSS13025 virus (Figure 4A). Likewise, MR766 virus grew to 6-, 9-, and 35-times higher titers than FSS13025 virus after 1, 2, and 3 days p.i. (Figure 4B). These results are consistent with the previous findings that African-lineage ZIKVs are more virulent and have higher growth capacity than Asian-lineage ZIKVs [15,29,30,31]. These observations may partially explain the higher titers of infectious MR766 virus and leaking transcription of MR766 RNA upon DNA transfection compared to FSS13025 virus (Figure 2 and Figure 4). 

### 3.4. Validation of Infectious ZIKV by the Identification of Genetic Markers

**Figure 5:**
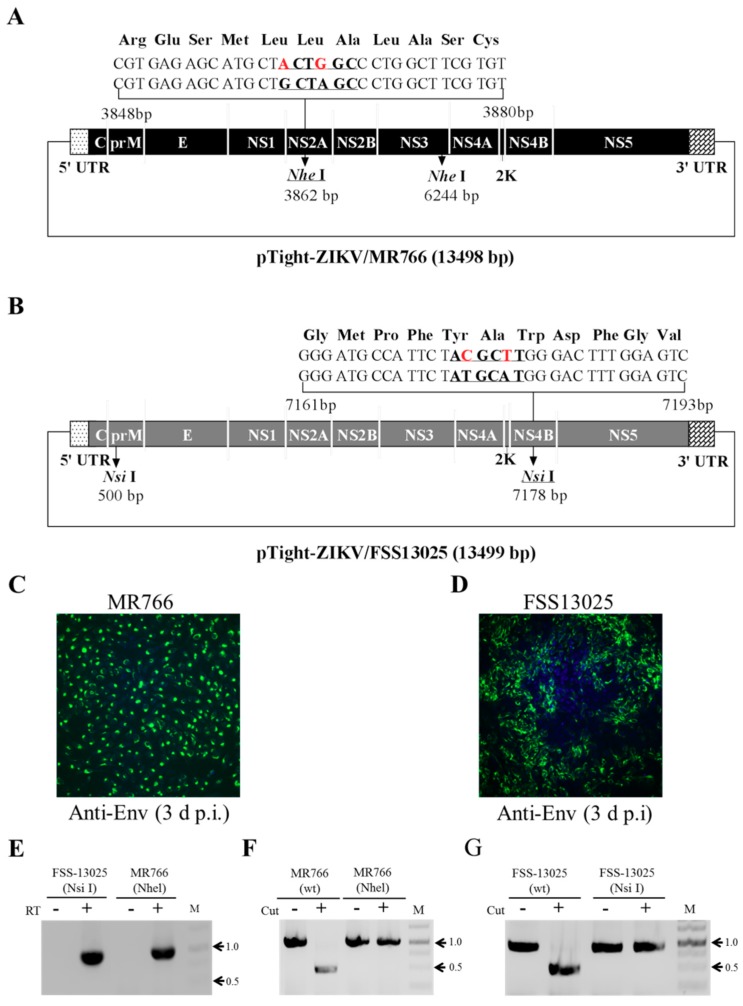
Confirmation of cDNA-derived ZIKV by detection of genetic markers. (**A**) Diagram of the pTight-ZIKV/MR766 DNA containing two nucleotide mutations as genetic markers. The *Nhe* I site at the nucleotide 3862 of the MR766 cDNA was mutated by changing two nucleotides (red) that do not alter amino acids (shown on the top). (**B**) Diagram of the pTight-ZIKV/FSS13025 DNA containing two nucleotide mutations at the ^7178^*Nsi* I site of the FSS13025 cDNA as genetic markers. (**C**,**D**) Validation of cDNA-derived infectious ZIKV by IFA. The genetic markers-harboring pTight-ZIKV/MR766 and pTight-ZIKV/FSS13025 DNAs were co-transfected into Vero cells as described in Figure 3. The supernatants of the DNA-transfected cells were used for infection of Vero cells. At 3 d p.i., the expression of the E protein of MR766 (**C**) or FSS13025 (**D**) was detected by IFA. The images of (**C**,**D**) were taken under 200× magnification. The vRNAs were extracted from the supernatants using a Qiagen viral RNA isolation kit and used for reverse transcription (RT, indicated by +). The RT products were amplified by PCR using vRNA without RT as controls (**E**). The RT-PCR products from MR766 vRNA were digested with the restriction enzyme *Nhe* I (**F**), whereas the RT-PCR products of FSS13025 were cut with *Nsi* I (**G**). Wild type MR766 and FSS13025 RT-PCR products were used as controls.

To discriminate cDNA-derived recombinant virus from potentially contaminated ZIKV, one of the two *Nhe* I sites present in the MR766 cDNA was mutated by introducing silent nucleotide mutations as genetic markers for the cDNA-derived ZIKV (Figure 5A). Similarly, one of the two *Nsi* I sites found in the FSS13025 cDNA was mutated (Figure 5B). Upon DNA transfection, resulting infectious MR766 (Figure 5C) and FSS13025 virus (Figure 5D) were confirmed by RT-PCR (Figure 5E) and digestion with restriction enzymes *Nhe* I for MR766 virus (Figure 5F) and *Nsi* I for FSS13025 virus (Figure 5G). The RT-PCR DNAs amplified from wild type but not recombinant viruses were digested with *Nhe* I (MR766) or *Nsi* I (FSS13025). The presence of genetic markers introduced into viral cDNA confirms the authenticity of the cDNA-derived ZIKV. 

### 3.5. Stability of Infectious ZIKV cDNA in E. coli

**Figure 6:**
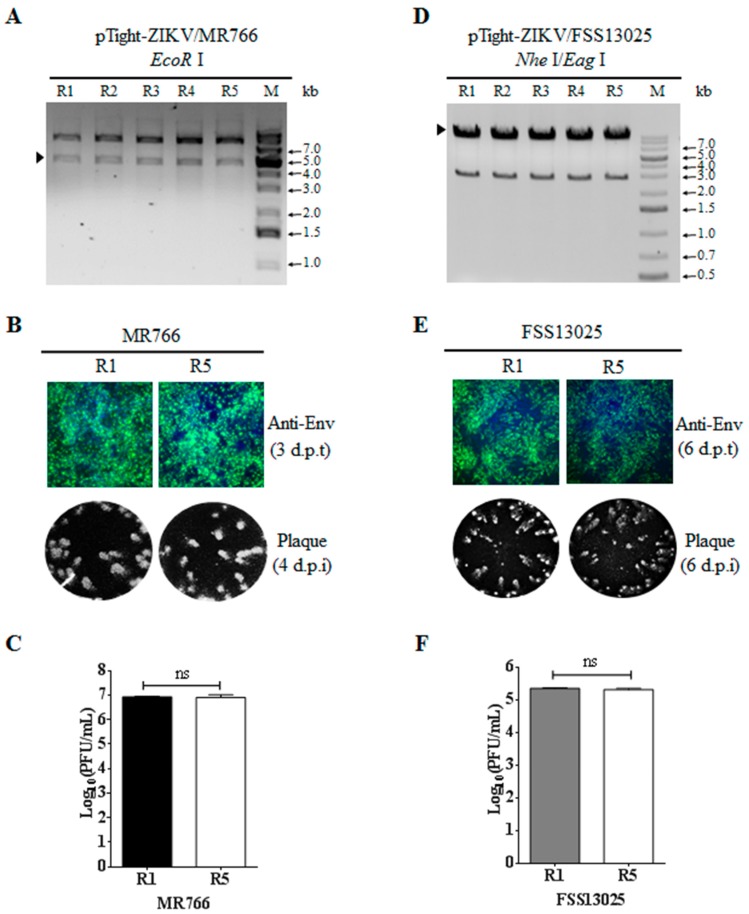
Determination of genetic stability of infectious ZIKV cDNA clones in *E. coli*. (**A**) Analysis of the pTight-ZIKV/MR766 DNA stability by *EcoR* I digestion. The pTight-ZIKV/MR766 plasmid was sequentially transformed to and amplified in *E. coli* for five consecutive rounds (R1 to R5). The Plasmid DNA purified from each round was digested with *EcoR* I and analyzed by electrophoresis on 1% of agarose gel. The DNA fragment released from ZIKV/MR766 cDNA upon *EcoR* I digestion is indicated by a solid arrow on the left. GeneRuler 1 kb plus DNA ladder (Fisher Scientific, Waltham, MA, USA) is used as DNA size marker (M) shown on the right. (**B**) Confirmation of the pTight-ZIKV/MR766 DNA stability by functional analysis in Vero cells. 1 μg of pTight-ZIKV/MR766 DNAs purified from the first (R1) and fifth (R5) rounds of amplification were co-transfected with 1 μg of pTet-On into Vero cells with the addition of 1 μg/mL of doxycycline to cell culture medium. At 3 d p.t., the viral E protein expression was detected by IFA (up panel, 200× magnification), whereas infectious virus in the supernatant was measured by a plaque assay (lower panel). (**C**) Comparison of infectious virus titers resulting from the pTight-ZIKV/MR766 DNA between R1 and R5. (**D**) Analysis of genetic stability of the pTight-ZIKV/FSS13025 DNA by digestion with restriction enzymes *Nhe* I and *Eag* I. The FSS13025 cDNA released from the pTight-ZIKV/FSS13025 DNA upon digestion with *Nhe* I and *Eag* I (Figure 1C) is indicated by a solid arrow on the left. (**E**) Validation of cDNA-derived ZIKV/FSS13025 upon transfection with R1 and R5 pTight-ZIKV/FSS13025 DNA. Experiments were the same as B except R1 and R5 pTight-ZIKV/FSS13025 DNAs were used. (**F**) Comparison of infectious ZIKV/FSS13025 tiers resulting from R1 and R5 DNA transfection. Values represent the means ± standard deviations (SD) from three independent experiments. Statistical significance was analyzed by Student’s *t*-test. ns indicates no significant difference.

Previous studies by others suggested that infectious ZIKV cDNA is genetically unstable during amplification in bacteria. To determine the genetic stability of our infectious MR766 and FSS13025 cDNA clones based on the Tet-On system, the pTight-ZIKV/MR766 and pTight-ZIKV/FSS13025 plasmids were sequentially transformed to and amplified in *E. coli* up to 5 rounds, similar to the study described previously by others [18]. Plasmid DNA extracted at each round of transformation and amplification was digested with restriction enzyme *EcoR* I (for MR766 cDNA) or *Nhe* I and *Eag* I (for FSS13025 cDNA). The patterns of restriction enzyme digestion remained the same between different rounds of transformation and amplification in *E. coli* (Figure 6A,D), suggesting that there was no large DNA deletion or insertion during plasmid DNA amplification in *E. coli.* More importantly, transfection of Vero cells with plasmid DNA purified from the first round (R1) and fifth round (R5) of transformation resulted in similar levels of the E protein expression and comparable plaque size and numbers (Figure 6B,E) as well as the same titers of infectious ZIKV (Figure 6C,F). These results demonstrate the genetic stability of the infectious ZIKV cDNA cloned into the Tet-inducible vector modified in the lab.

## 4. Discussion

In the present study, we have developed a robust reverse genetics system for production and genetic manipulation of infectious ZIKV using the Tet-inducible (Tet-On) gene expression strategy [32]. The Tet-inducible ZIKV reverse genetics system is superior in many respects to previous ones based on the in vitro T7/SP6 transcription [18,25], in vitro DNA ligation or recombination from multiple DNA fragments [20,21,26], and the insertion of the intron sequence into the *NS1* or *NS5* gene of ZIKV cDNA [16,19]. The Tet-responsive *P*_tight_ promoter used in our ZIKV cDNA constructs is able to drive the transcription of infectious ZIKV RNA in the cell upon DNA transfection. In the case of T7/SP6 promoter-based vectors, in vitro transcription of infectious ZIKV RNA by T7/SP6 RNA polymerases requires the use of a 7-methyl guanosine nucleotide (m7G(5′)ppp(5′)G) or cap structure derivative [18,25]. The low efficiency of incorporation of the cap structure into T7/SP6 transcripts could affect the quantity of infectious RNA transcripts. Also, RNA transfection is less efficient than DNA transfection. Similarly, in vitro DNA ligation or recombination from multiple subgenomic cDNAs for construction of infectious ZIKV cDNA are laborious, time-consuming, and very inefficient [20,21,26]. Comparing to in vitro RNA transcription methods, our DNA-based vectors for the production of infectious ZIKV in the cell are robust, convenient, and cost-effective. More importantly, the Tet-inducible production of infectious ZIKV from the DNA-based vector transfected into the cell is highly efficient and can be induced by the addition of doxycycline. Although mammalian gene expression vectors were successfully used for production of infectious ZIKV, they require the insertion of the intron sequence into the NS1 or NS5 gene in order to be genetically stable in *E. coli* [16,19]. Subsequent production of infectious ZIKV depends on the splicing of RNA transcripts to remove the intron sequence inserted into the *NS1* or *NS5* gene. It is not clear whether the intron sequence inserted into the ZIKV cDNA will affect the efficiency of virus production in the cell. It was recently found that dengue virus NS5 protein is predominantly localized in the nucleus and is able to suppress RNA splicing by binding to core components of the spliceosome [33]. Although the ZIKV NS5 protein is also exclusively localized in the nucleus [34], its role in RNA splicing has not been determined. This appeared not to be a problem for production of more infectious ZIKV like MR766 strain from its cDNA in the cell given the facts that leaky RNA transcription from the pTight-ZIKV/MR766 DNA was sufficient to produce infectious virus (Figure 2A and Figure 3A). This could be partially due to higher growth capacity of ZIKV/MR766 in the cell (Figure 4). A higher efficiency of RNA transcription is likely required for the production of those ZIKV strains with lower replication efficiency like the FSS13025 strain. Unlike MR766, FSS13025 virus production from its cDNA requires co-transfection with the pTet-On vector and the addition of doxycycline (Figure 2B and Figure 4). In this aspect, the newly developed reverse genetics system through this study will be particularly useful for pathogenesis study and genetic manipulation of clinical isolates of ZIKV. Inducible production of the recombinant virus of clinical ZIKV isolates will be suitable for the study of viral pathogenesis in transgenic animals, which can be controlled by treatment with doxycycline. For instance, transgenic mice carrying the full-length cDNA of ZIKV under the control of pTight promoter can be used for studying congenital ZIKV infection by administrating animals with doxycycline. For genetic studies, robust production of infectious ZIKV as shown by our system may facilitate the study of virus–host interaction and the development of attenuated viruses. This may explain why the intron-based DNA vector for transposon mutagenesis of the ZIKV/MR766 resulted in fewer transposon insertion mutants [35]. Future investigations are warranted to determine if the Tet-inducible ZIKV reverse genetics system will be more efficient than the intron-based system for genome-wide analysis of viral sequence and proteins in viral replication and pathogenesis. Taken together, our findings suggest that the newly developed Tet-inducible ZIKV cDNA vector is highly efficient and robust for the production of infectious ZIKV, especially for attenuated viruses or virus strains with lower replication efficiency. This new DNA-based ZIKV reverse genetics system will facilitate genetic analysis and genetic manipulation of various ZIKV strains regardless of their replication efficiency. 

Over the years, the development of reverse genetics systems for flaviviruses has been proven difficult due to their genetic instability in and/or toxicity to *E. coli* during cDNA cloning [36]. The underlying molecular mechanism of viral genetic instability and/or toxicity in *E. coli.* remains elusive. In general, it is believed that cryptic bacterial promoters present in viral cDNA or promoters used to drive the transcription of viral RNA are active in *E. coli,* resulting in the expression of toxic peptides or proteins [22,24,36]. To circumvent the difficulties in ZIKV cDNA cloning, several strategies have been used, including but not limited to the use of low-copy plasmids as vectors for in vitro RNA transcription; the separate cloning of the full-length cDNA as subgenomic DNA fragments in conjunction with in vitro DNA ligation or assembly; the insertion of a spacer sequence such as the intron to disrupt the open-reading frame [36]; and the silencing of cryptic prokaryotic promoters [23]. The immediate early CMV promoter was previously shown to be able to initiate gene expression in *E. coli* [37]. This may explain the viral toxicity observed during the MR766 cDNA cloning to a vector under the control of a CMV promoter, resulting in large deletions in the *NS1* to *NS3* coding regions [16]. To avoid this potential problem, we decided to choose the Tet-responsive *P*_tight_ promoter, which contains the minimal CMV promoter sequence of 59 nucleotides and can inducibly initiate the transcription of infectious ZIKV RNA. Additionally, we have replaced the high copy-number origin of replication present in the pTRE-Tight vector with the low-copy-number origin sequence of the vector pACYC177, which was successfully used for cloning of the first ZIKV cDNA [18]. It is most likely that the combination of the *P*_tight_ promoter and the low-copy-number origin of replication eliminated viral toxicity and, therefore, confer genetic stability of the pTight-ZIKV/MR766 and pTight-ZIKV/FSS13025 DNA vectors in *E. coli* (Figure 6). The modified pTight-ZIKV entry vectors (Figure 1) can be used for the construction of infectious cDNAs of all other ZIKV strains.
